# Colchicine Poisoning: A Rare Case

**DOI:** 10.7759/cureus.48933

**Published:** 2023-11-16

**Authors:** Andreia Amaral, Diogo Ferreira da Silva, Maria Beatriz Sampaio, Catarina Salvado

**Affiliations:** 1 Internal Medicine, Centro Hospitalar Universitário de Lisboa Central, Lisbon, PRT

**Keywords:** treatment-related toxicity, rare cause of pleural effusion, colchicine poisoning, substance-related disorders, case reports

## Abstract

Intoxication by colchicine is rare, and its rapid recognition is crucial, as severe toxicity or death is reported in 10% of cases.

Here, we present the case of a 50-year-old female admitted to the emergency department 24 hours after ingesting 10 mg of colchicine. Upon examination, she was conscious and hemodynamically stable. Analytically, she exhibited leukocytosis with neutrophilia and an elevation of lactate dehydrogenase (LDH). She was initiated on fluid therapy and transferred to the intermediate care unit of medicine. On the third day of hospitalization, she developed anterior chest pain, reduced breath sounds in the right hemithorax, and dullness on percussion. Arterial blood gas analysis showed partial respiratory failure, and chest X-rays and a computed tomography (CT) scan revealed a right-sided pleural effusion. The likely diagnosis was pleural effusion secondary to cardiac dysfunction due to colchicine intoxication.

This case aims to describe the potential toxic effects of colchicine in cases of overdose and to reflect on ways to reduce its morbidity and mortality.

## Introduction

Colchicine is an alkaloid primarily used in the treatment of certain inflammatory diseases such as gout, familial Mediterranean fever, and pericarditis [1-3]. It is rapidly absorbed orally and undergoes extensive first-pass metabolism. It is primarily metabolized by the liver, subject to enterohepatic recirculation, and excreted through the renal route. This substance has a narrow therapeutic index, with no clear distinction between nontoxic, toxic, and lethal doses. Generally, colchicine toxicity is dose-dependent, with reported cases of high fatality rates following acute ingestion of more than 0.5 mg/kg and reported lethal doses ranging from 7 to 26 mg [1,4].

The toxicity of colchicine is an extension of its mechanism of action, as it binds to tubulin, disrupting the microtubular network. As a result, cells exhibit changes in protein synthesis, reduced endocytosis and exocytosis, altered cellular morphology, decreased cellular motility, disruption of mitosis, and impairment of cardiac myocyte conduction and contractility, leading to dysfunction and multi-organ failure [4,5].

Although colchicine poisoning is rare, it can occur accidentally or intentionally. Early recognition is crucial for its treatment and stabilization, as severe toxicity or death has been described in 10% of cases [6].

We report the case of a patient with intoxication from 10 mg of colchicine.

## Case presentation

A 50-year-old female was admitted to the emergency department with complaints of nausea and vomiting 24 hours after intentionally ingesting 10 mg of colchicine. Her personal medical history included an ongoing psychotic condition under study, and she was not taking any regular medications. She denied any known allergies, alcohol or tobacco use, or a history of substance abuse.

Upon physical examination in the emergency department, she was conscious and oriented. Her vital signs showed a blood pressure of 117/77 mmHg, a heart rate of 86 beats per minute (bpm), and a tympanic temperature of 36°C. Abdominal palpation revealed tenderness in the hypogastric region, with no other significant findings.

Analytically, the following findings are notable: hemoglobin of 16 g/dL, leukocytosis of 11.18 × 10^9^/L with neutrophilia (83.8%), platelets of 189 × 10^9^/L, normal renal function with urea of 37 mg/dL and creatinine of 0.92 mg/dL, slight elevation in aspartate aminotransferase (AST) of 86 U/L, elevated lactate dehydrogenase (LDH) of 692 U/L, and normal electrolyte levels; urinalysis revealed no significant abnormalities. An electrocardiogram showed sinus rhythm (96 bpm) with no ventricular repolarization abnormalities. The chest X-ray did not reveal any acute pleuroparenchymal findings.

She was assessed by a psychiatrist who described delusional self-referential ideation, which was to be further clarified after the resolution of the acute condition.

The patient was initiated on intensive intravenous fluid therapy and antiemetic therapy with metoclopramide and was transferred to the intermediate care unit for monitoring.

On the third day of hospitalization, she developed anterior chest pain, and the physical examination revealed asymmetric lung auscultation with decreased breath sounds in the right hemithorax and dullness on percussion. She presented with partial respiratory failure, with a partial pressure of oxygen (pO2) of 70.4 mmHg, requiring low-flow oxygen therapy.

**Figure 1:**
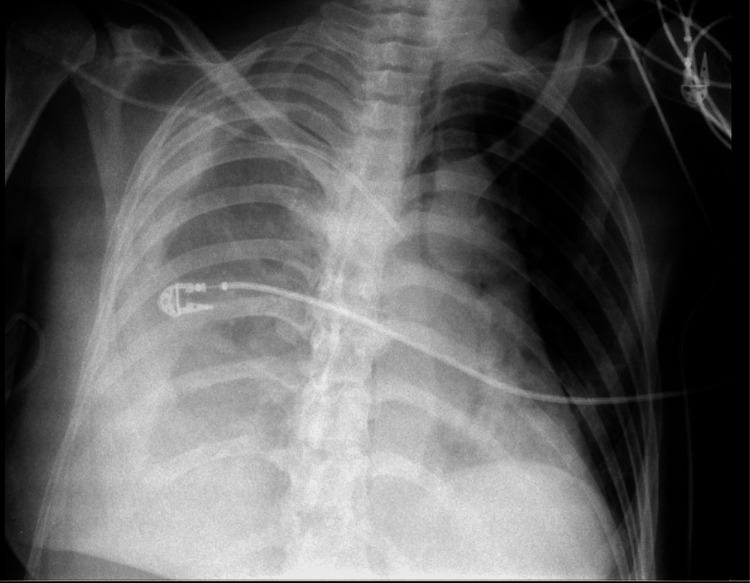
Anteroposterior chest X-ray showing reduced transparency of the right hemithorax, suggestive of pleural effusion

**Figure 2:**
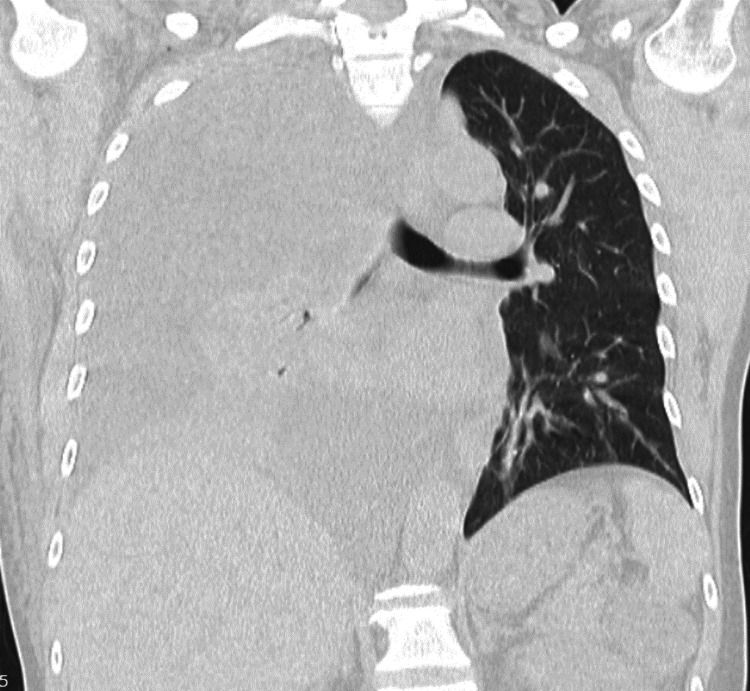
Chest computed tomography with a large right-sided pleural effusion causing contralateral mediastinal shift and nearly complete passive atelectasis of the right lower lobe

A chest X-ray showed a right-sided pleural effusion (Figure 1), and a chest computed tomography (CT) described a large right-sided pleural effusion with a loculated component of watery density, causing contralateral mediastinal shift and nearly complete passive atelectasis of the right lower lobe (Figure 2). There was also a minor left pleural effusion, leading to atelectasis of adjacent lung segments. In the lung parenchyma, ground-glass opacities with some areas tending toward consolidation were identified in the apical segment of the right upper lobe. Furthermore, pulmonary arterial trunk dilation, measuring 32 mm, was suggestive of pulmonary arterial hypertension, and there was a slight pericardial effusion. A transthoracic echocardiogram (no image available) revealed mild mitral and tricuspid regurgitation, preserved global and segmental systolic function, a non-dilated inferior vena cava (11 mm) with a collapsibility index not exceeding 50%, and the presence of a loculated right retro-auricular pericardial effusion.

Based on the described results and an N-terminal pro-B-type natriuretic peptide (NT-proBNP) level of 738.7 pg/mL, pleural effusion was assumed to be secondary to cardiac dysfunction in the probable context of colchicine intoxication. Intravenous diuretic therapy with furosemide 20 mg every six hours was initiated.

Peripheral venous blood analysis (Table 1) initially indicated an increase in serum creatinine with maintained urine output and a positive fluid balance, suggesting a hypervolemic prerenal acute kidney injury.

**Table 1:** Laboratory values of the patient according to days after colchicine intoxication Upward arrows indicate an increase in value, and downward arrows indicate a decrease. INR: international normalized ratio, NT-proBNP: N-terminal pro-B-type natriuretic peptide

	D3	D4	D5	D6	D7	D8	D9	D12	Normal range
Hemoglobin (g/dL)	16.6	14.9	14.1	13.3	14.2	13.9	14.6	15	12-15
Leukocytes (×10^9^/L)	14.83↑	7.08	2.57↓	3.2↓	4.26↓	6.63	9.13↑	15.63↑	4.5-11
Platelets (×10^9^/L)	134↓	82↓	50↓	46↓	44↓	59↓	109↓	209	150-450
INR	1.64↑	1.35	0.98	1.01	-	0.8	0.8	1.07	0.80-1.20
Urea (mg/dL)	62↑	110↑	129↑	135↑	140↑	147↑	102↑	82↑	21-43
Creatinine (mg/dL)	2.66↑	2.83↑	2.34↑	2.21↑	1.94↑	1.52↑	1.12↑	0.91	0.57-1.11
Aspartate aminotransferase (U/L)	-	167↑	117↑	191↑	215↑	91↑	60↑	62↑	5-34
Alanine aminotransferase (U/L)	38	39	33	151↑	258↑	194↑	159↑	133↑	0-55
Gamma-glutamyl transferase (U/L)	-	25	31	72↑	137↑	110↑	117↑	90↑	9-36
Alkaline phosphatase (U/L)	160↑	157	84	75	90	83	87	82	40-150
Lactate dehydrogenase (U/L)	1,734↑	1,879↑	1,136↑	688↑	561↑	369↑	333↑	312↑	125-220
Sodium (mEq/L)	141	140	137	138	138	138	139	133↓	136-145
Potassium (mEq/L)	3.9	3.4	4	3.7	3.6	3.4	3.7	4.3	3.50-5.10
Chloride (mEq/L)	104	108	110	106	102	99	97↓	93↓	98-107
Magnesium (mg/dL)	1.55	2.66↑	2.72↑	2.21	2.09	1.97	2	1.93	1.60-2.60
C-reactive protein (mg/L)	80.7	114.4	74.3	68.2	62.5	53.8	54.7	10.2	<5
Total creatine kinase (U/L)	384↑	492↑	449↑	299↑	238↑	-	181↑	862↑	29-168
NT-proBNP (pg/mL)	-	-	-	738.7↑	-	-	-	133.4	<249
Uric acid (mg/dL)	-	-	-	9.1↑	10.6↑	-	-	-	2.60-6

**Figure 3:**
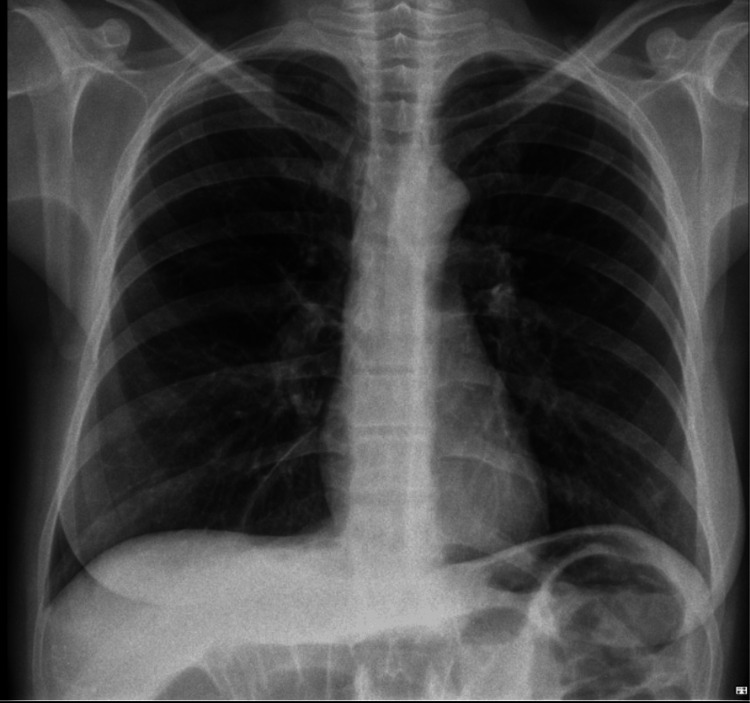
Posteroanterior chest X-ray without alterations

In the following days, there was progressive improvement in renal function and complete resolution of the pleural effusion (Figure 3).

From a hematologic perspective, three days after intoxication, the patient developed progressive thrombocytopenia, with a minimum platelet count of 44 × 10^9^/L reached on the seventh day, without any signs of bleeding disorders. Leukopenia and lymphopenia were also observed from the fifth day, with a return to normal values on the eighth day and subsequent leukocytosis. From a gastrointestinal standpoint, the patient experienced diarrhea on the fourth day after ingesting colchicine, which resolved within two days after the introduction of loperamide. In the initial two days of hospitalization, the patient had intermittent fever spikes, but no specific causative agent was isolated, so antibiotic therapy was not initiated. On the 10th day of hospitalization, the patient developed alopecia. With clinical stability, the patient was discharged and referred for follow-up to the psychiatry and internal medicine clinics.

## Discussion

In cases of acute colchicine poisoning, the use of activated charcoal and, potentially, gastric lavage should be considered, particularly when a large quantity of the drug has been ingested within the preceding 60 minutes [4,7]. If a patient remains asymptomatic after 24 hours of observation in the emergency department, discharge can be considered [6]. In the previously described case, the ingestion of colchicine had occurred more than 24 hours before admission, and the patient was symptomatic, so supportive treatment was initiated.

Colchicine intoxication typically manifests in three sequential and overlapping phases: the gastrointestinal phase, the multiple organ dysfunction phase, and the recovery phase. The gastrointestinal phase (10-24 hours post-ingestion) is characterized by symptoms resembling gastroenteritis, including nausea, vomiting, diarrhea, and abdominal discomfort. Patients may experience hypovolemia and leukocytosis during this phase. In the multiple organ dysfunction phase (24 hours to seven days post-ingestion), multiple organ failure and rapidly progressive sepsis can occur due to the infectious risk associated with the development of pancytopenia. In the recovery phase (7-21 days post-ingestion), it is common to observe alopecia. Generally, patients experience complete recovery [3,4].

Early recognition and management are crucial in colchicine intoxication to mitigate the potential progression to the severe multi-organ dysfunction phase. The described case illustrates the importance of prompt diagnosis and treatment in such situations to achieve a favorable outcome.

In the case of the described patient, it was unexpected that gastrointestinal symptoms, such as nausea, vomiting, and diarrhea, appeared relatively later (between the second and fourth day), as these symptoms are typically expected to manifest earlier. Gastrointestinal symptoms can be prominent in colchicine intoxication due to prolonged exposure of the intestinal mucosa to high drug concentrations caused by enterohepatic circulation [1,2]. However, in this case, the gastrointestinal symptoms were moderate and completely reversed after the introduction of loperamide.

The patient's progression of symptoms aligns with other reported cases, where thrombocytopenia was observed on the third day and leukopenia on the fifth day, followed by later recovery and leukocytosis on the ninth day. It is essential to monitor the patient's temperature profile because fever can be a manifestation of colchicine toxicity or a sign of infectious complications, which, in these cases, can quickly lead to septic shock, especially when there is pancytopenia. If significant bone marrow involvement occurs, the use of growth factors or blood products is recommended [1,6]. However, this was not necessary in the case of the patient presented, given the rapid response and absence of signs or symptoms suggestive of infection.

Liver toxicity is rare [1,2], and as observed in this case, only mild hepatocellular cholestasis occurred between the fourth and ninth days, without hyperbilirubinemia.

On the fourth day of hospitalization, the patient developed partial respiratory failure with evidence of pleural effusion. Respiratory involvement is described in about one-third of cases, presenting with hypoxemia and diffuse interstitial and alveolar edema on chest X-ray [1,2], which was not observed in this case. Considering the right pleural effusion of watery content and the elevated NT-proBNP, the most likely diagnosis was drug-induced heart failure. The mechanism of cardiac dysfunction is not entirely clear, but the drug may have a direct toxic effect on myocardial function, while a postulated specific vasodilatory effect could explain the sometimes observed hyperkinetic state [1,2].

Renal involvement is often multifactorial, either due to hypovolemia in cases with prominent gastrointestinal symptoms or secondary to multi-organ failure [8].

Alopecia, as typically described, appeared on the 10th day. Hair loss is usually confined to the scalp, and in most cases, it is reversible, with hair regrowth occurring within 3-12 weeks after discontinuation of the drug [1,2].

In this case, notable is the absence of central nervous system involvement, such as changes in mental status, transverse myelitis, and ascending paralysis, as described in other cases [1,2].

In the treatment of colchicine poisoning, considering its tissue distribution, plasma exchange and hemoperfusion are not effective [9]. Supportive treatments, including the administration of granulocyte colony-stimulating factor, form the basis of treatment. Specific experimental treatment with Fab fragment antibodies is being studied, but it is not currently available [4,7].

A limitation of this case is the inability to determine the serum colchicine level, with the diagnosis being made based on the clinical history. Given the frequency of colchicine use, further studies on its effects and prognostic factors for toxicity are essential and warranted.

## Conclusions

This case emphasizes the importance of active monitoring of symptomatic patients due to the risk of developing multi-organ failure, which typically occurs within 24-36 hours after ingestion, as well as subsequent infectious or hemorrhagic complications.
